# Effect of sulfate addition on carbon flow and microbial community composition during thermophilic digestion of cellulose

**DOI:** 10.1007/s00253-020-10546-7

**Published:** 2020-03-26

**Authors:** Nina Lackner, Andreas O. Wagner, Paul Illmer

**Affiliations:** Department of Microbiology, Universität Innsbruck, Technikerstraße 25d, 6020 Innsbruck, Austria

**Keywords:** Sulfate reducing bacteria, Methanogens, Inhibition, Community composition, Hydrogen sulfide, Acetate

## Abstract

Substrates with high sulfate levels pose problems for biogas production as they allow sulfate reducing bacteria to compete with syntrophic and methanogenic members of the community. In addition, the end product of sulfate reduction, hydrogen sulfide, is toxic and corrosive. Here we show how sulfate addition affects physiological processes in a thermophilic methanogenic system by analyzing the carbon flow and the microbial community with quantitative PCR and amplicon sequencing of the 16s rRNA gene. A sulfate addition of 0.5 to 3 g/L caused a decline in methane production by 73–92%, while higher sulfate concentrations had no additional inhibitory effect. Generally, sulfate addition induced a shift in the composition of the microbial community towards a higher dominance of *Firmicutes* and decreasing abundances of *Bacteroidetes* and *Euryarchaeota*. The abundance of methanogens (e.g., *Methanoculleus* and *Methanosarcina*) was reduced, while sulfate reducing bacteria (especially *Candidatus Desulforudis* and *Desulfotomaculum*) increased significantly in presence of sulfate. The sulfate addition had a significant impact on the carbon flow within the system, shifting the end product from methane and carbon dioxide to acetate and carbon dioxide. Interestingly, methane production quickly resumed, when sulfate was no longer present in the system. Despite the strong impact of sulfate addition on the carbon flow and the microbial community structure during thermophilic biogas production, short-term process disturbances caused by unexpected introduction of sulfate may be overcome due to the high resilience of the engaged microorganisms.

## Introduction

The anaerobic digestion of organic waste of changing composition and quantity for biogas production involves the risk of introducing undesirable substances to the system, which endanger optimal process performance (Illmer and Gstraunthaler 2009; Wagner et al. 2014). One of these substances is sulfate (SO_4_^2−^), which can be introduced in the reactor when digesting wastes from the food industry, particularly from the production of alcohol, yeast, citric acid and edible oils, and the paper industry (Colleran et al. 1995). The input of high levels of SO_4_^2−^ into biogas fermenters results in lower methane (CH_4_) production rates and the evolution of hydrogen sulfide (H_2_S), which causes odors and corrosion. In the presence of SO_4_^2−^ as a terminal electron acceptor, SO_4_^2−^ reducing bacteria (SRB) compete with syntrophic bacteria and methanogens for their common substrates (lactate, acetate, propionate, butyrate, and H_2_) and thereby produce cytotoxic H_2_S (Muyzer and Stams 2008). Data on inhibiting SO_4_^2−^ levels vary largely as the effect of SO_4_^2−^ in an anaerobic digestion process depends on various factors, such as the type of reactor, the operation temperature, the used substrates, the pH, and the native degrading community (Colleran and Pender 2002; Chen et al. 2008). Nevertheless, it is generally agreed that the ratio of available carbon to SO_4_^2−^ is more decisive than the SO_4_^2−^ concentration itself and that at a chemical oxygen demand (COD) to SO_4_^2−^ ratio of 10 or larger methanogenesis is not inhibited, while below this value both, successful and unsuccessful anaerobic digestion may occur (Hulshoff Pol et al. 1998).

SRB affect the anaerobic community at multiple degradation levels, although it is assumed that SRB cannot compete with fast-growing acidogenic, fermenting organisms (Chen et al. 2008). From a thermodynamic point of view, SRB should out-compete acetogenic and methanogenic organisms for hydrogen (H_2_), acetate, propionate, and butyrate (Stams et al. 2005). In reality, however, the dominance of SRB is, depending on the substrate, less distinct, as acetogens or methanogens may be advantageous concerning their pH and temperature optimum, sensitivity to H_2_S toxicity, initial abundance, or growth rate (Colleran and Pender 2002; Paulo et al. 2015; Chen et al. 2008). Therefore, experimental data on the competition of SRB with acetogenic and methanogenic organisms are often contradictory (Chen et al. 2008). Nevertheless, it was mostly found that SRB outcompete their opponents for H_2_ and propionate, while the outcome of the competition for acetate and butyrate is rather unclear (Colleran et al. 1995; Paulo et al. 2015; Chen et al. 2008). Under SO_4_^2−^ limiting conditions, SRB can grow as acetogens, meaning that they may be abundant in biogas reactors even after long periods of SO_4_^2−^ absence (Visser et al. 1993). The toxicity of H_2_S is dependent on the pH, as unionized H_2_S can diffuse through the cell membrane and cause damage to proteins, coenzymes and can interfere with the assimilatory sulfur metabolism (Chen et al. 2008). The susceptibility to sulfide inhibition differs between the trophic groups and can be sorted as follows: hydrogenotrophic methanogens < hydrogenotrophic SRB < propionate oxidizing SRB < acetotrophic methanogens < syntrophic propionate oxidizing bacteria < acetate oxidizing SRB (Maillacheruvu and Parkin 1996). The inhibitory levels for dissolved sulfides in the literature range from 100 to 800 mg/L (Chen et al. 2008).

Most research on SO_4_^2−^ in anaerobic digestion is descriptively investigating real-life substrates at mesophilic conditions. The present study combines a defined substrate with an undefined mixed methanogenic community at thermophilic conditions to investigate competition-related and inhibitory effects of SO_4_^2−^ addition to a reproducible system. In a first step, we examined the effects of various SO_4_^2−^ levels on the applied methanogenic system regarding CH_4_ production, SO_4_^2−^ reduction, microbial community composition, and abundance of the relevant physiological groups. In a second step, we focused on SO_4_^2−^ induced shifts in carbon flow over a period of 4 weeks of anaerobic digestion.

## Material and methods

### Experimental design

The investigation was composed of two main experiments and a viability test. The aim of the first experiment was to determine the inhibition threshold of SO_4_^2−^ on the applied methanogenic system. The tested SO_4_^2−^ concentrations were 0, 0.5, 1.5, 3, and 5 g SO_4_^2−^/L. The variants were incubated for 4 weeks at 50 °C and evaluated concerning CH_4_, carbon dioxide (CO_2_) and H_2_ production, reduction of SO_4_^2−^, pH, production of H_2_S, abundance of methanogens and SRB, as well as shifts in the microbial community composition (see below). In a second experiment, the variants supplemented with 0 and 3 g SO_4_^2−^/L were repeated and analyzed more comprehensively regarding changes in the carbon flow. To examine if methanogens and SRB in the SO_4_^2−^ containing samples were still alive on day 28, a viability test was performed. For this purpose, 0.1 mL of a parallel from day 28 containing 3 g SO_4_^2−^/L was used to inoculate fresh media with 0 or 3 g SO_4_^2−^/L in triplicate and incubated as described above experiment. Increasing CH_4_ and H_2_S concentrations were used as viability proof for methanogens and SRB, respectively.

### Medium and inoculum

The anaerobic medium contained per liter: 1 g NaCl, 0.4 g MgCl_2_ × 6 H_2_O, 0.5 g KCl, 0.2 g KH_2_PO_4_, 0.15 g CaCl × 2H_2_O, 0.5 g l-cysteine, 1 mL resazurin solution (1.15% w/v), 1 mL trace element solution (Wagner et al. 2019), 10 g carboxymethylcellulose, 2 g yeast extract, 3 g peptone from casein, 8.4 g NaHCO_3_, and the intended amount of Na_2_SO_4_^2−^ (0, 0.5, 1.5, 3, or 5 g SO_4_^2−^/L). The pH was adjusted to 7.75 and the medium autoclaved. Subsequently, 1 mL vitamin solution and 2 mL reducing agent (0.24 g NaS_2_/L final concentration) were added aseptically; the medium was aliquoted at 50 mL in 120-mL serum flasks and flushed with N_2_/CO_2_ (70:30) to ensure anaerobic conditions. The flasks were inoculated with 5 mL 1:5 diluted fermenter sludge from a thermophilic plug flow fermenter treating organic waste in Roppen, Tirol (Austria), as described elsewhere (Wagner et al. 2014).

### Biochemical analysis

To calculate the total biogas production the overpressure was measured with a digital precision manometer (GDH 200-13, GREISINGER electronic, Germany) and normalized to ambient pressure (data from Zentralanstalt für Meterologie und Geodynamik, Austria). The gas composition was analyzed on a Shimadzu GC2010, as described in Wagner et al. (2011). Gas samples were diluted with N_2_ prior the measurement to protect the column of the gas chromatograph (GC) from high H_2_S concentrations. CO_2_ and H_2_ concentrations were quantified via thermal conductivity detector, while CH_4_ concentrations were measured with a flame ionization detector. Volatile fatty acids (VFAs) were determined in undiluted liquid samples following the procedure in (Wagner et al. 2017). The pH was measured with a glass electrode. For dissolved organic carbon (DOC) contents, liquid samples were diluted, filtered (filter papers 615 1/4, Macherey-Nagel, Germany), and measured with a TOC/NPOC analyzer (Shimadzu, Japan) according to manufacturer’s protocol. SO_4_^2−^ was measured via ion chromatography (Metrohm, Herisau, Switzerland) by using a Metrosep A-Supp 5-250 column at 35 °C and a 753 Suppressor Module (Metrohm, Herisau, Switzerland). The solvent, containing 3.2 mM Na_2_CO_3_, and 1 mM NaHCO_3_ was used at a flow rate of 0.7 mL/min. The COD was measured with a Nanocolor® COD 1500 test (Macherey-Nagel, Germany) according to the user guide.

### Carbon flow diagrams

To create the carbon flow diagrams, all measurable carbon fractions were converted into millimoles, considering temperature and pressure. CO_2_ and CH_4_ values represent the cumulative gas amounts including day 0. Dissolved CH_4_ and CO_2_ were not included in the diagrams, because they could not be determined analytically or mathematically, and due to their relatively low solubility at 50 °C (Diamond and Akinfiev 2003). Acetate, propionate, and butyrate amounts quantified via high-performance liquid chromatography were subtracted from the DOC values. Diagrams exclude inorganic carbon and microbial biomass.

### Gibbs free energy calculations

To assess the thermodynamic properties of the common reactions under the prevailing conditions, biochemical data from day 14 were used together with the standard free Gibbs enthalpy (ΔG^0^) from (Amend and Shock 2001). H_2_S concentrations were estimated on basis of SO_4_^2−^ decrease, and the H_2_ partial pressure was assumed to be 0.002 mbar, which is the substrate threshold of SRB (Lovley et al. 1982).

### DNA extraction and quantification

For molecular analyses, 1 mL of culture fluid was centrifuged at 11,000×*g* for 10 min. Subsequently, 0.8 mL of supernatant was removed; the pellet was resuspended in the remaining fluid and used for DNA extraction. DNA extraction was done with a NucleoSpin® Soil kit (Macherey-Nagel, Germany) according to the manufacturer’s protocol. DNA was quantified using a Quant-iT™ PicoGreen™ dsDNA Assay Kit (Invitrogen, USA) and a multimode fluorometer Zenyth 3100 (Anthos, Austria).

### qPCR

The quantitative PCR (qPCR) to quantify methanogens and SRB targeted the functional genes methyl coenzyme M reductase α-subunit (mcrA) and dissimilatory sulfite reductase α-subunit (dsrA), respectively. The primers for the mcrA gene were mlas and mcrA-rev (Steinberg and Regan 2008) and as a standard served *Methanosarcina thermophila* (DSM 1825, DSMZ-German Collection of Microorganisms and Cell Cultures, Germany). The qPCR mix for mcrA contained per 20 μL 5.28 μL PCR grade water, 10 μL SensiFAST™ Probe No-ROX (Bioline, UK), 0.40 μL MgCl_2_ (50 mM), 0.38 μL of each primer (10 μM), 0.8 μL Enhancer (5×), and 2 μL template (diluted to 0.9–1.8 ng/μL). The primers used for SRB were dsrA_290F and dsrA_660R, and *Desulfovibrio vulgaris* (DSM 644, DSMZ, Germany) was used for the standards (Pereyra et al. 2010). The qPCR mix for dsrA contained per 20 μL 6.4 μL PCR grade water, 10 μL SensiFAST™ Probe No-ROX (Bioline, UK), 0.4 μL of each primer (10 μM), 0.8 μL Enhancer (5×), and 2 μL template (diluted to 0.9–1.8 ng/μL). The reactions were executed in a Rotor-Gene Q (Qiagen Bioinformatics, Germany) with an initial denaturation at 95 °C for 10 min (mcrA) or 3 min (dsrA), 45 (mcrA) or 50 (dsrA) cycles of 30 s (mcrA) or 40 s (dsrA) at 95 °C, 30 s at 66 °C (mcrA) or 60 °C (dsrA), and 30 s at 72 °C.

### Amplicon sequencing and data processing

Sequencing targeted the V4 region of the 16s rRNA gene using the primer pair 515f/806r (Apprill et al. 2015; Parada et al. 2016). To validate the performance of the entire sequencing process, a mock community was included in all steps beginning from DNA extraction until final data processing. The mock consisted of a ZymoBIOMICS Microbial Community Standard (Zymo Research, USA) mixed with a pure culture of *Methanosarcina thermophila* (DSM 1825, DSMZ, Germany) to also include archaeal DNA. In the first PCR step, the primers 515f/806r were used to attach an adapter sequence to the PCR fragment, conducting 30 cycles of 45 s at 95 °C, 45 s at 57 °C, and 60 s at 72 °C in a Flexcycler (analytikjena, Germany). The PCR mix contained per 25 μL reaction volume 12.5 μL Red Taq DNA Polymerase 2× Mastermix (VWR, USA), 0.8 μL 50 mM MgCl_2_, 0.5 μL of each primer, 1 μL enhancer (5×), and 1 μL template (diluted to 2.5 ng dsDNA/μL). In a second PCR step, individual barcodes for each sample were attached to the adapter sequence, running 7 cycles of 45 s at 95 °C, 45 s at 56 °C, and 90 s at 72 °C. Subsequently, the library was prepared with equal DNA amounts per sample to reach a final DNA concentration of 15 ng/μL. The library was cleaned up using a Hi Yield® Gel/PCR DNA Fragment Extraction Kit (Germany) and sent to Microsynth AG, Switzerland for sequencing (Illumina MiSeq 2 × 250 bp paired-end read).

### Data analysis and statistics

Sequencing data were processed with the software platform mothur v.1.39.5, 64 Bit executable (Schloss et al. 2009) using the SILVA database release 132 ribosomal SSU (release date 12.12.17) as a reference database (Quast et al. 2013; Yilmaz et al. 2014). Quality filtering of trimmed reads included screening for and removal of contigs longer than 300 bp, containing any ambiguities or more than 7 homopolymers. Chimera were identified and removed with VSEARCH 2.3.4 (Rognes et al. 2016). Unique sequences with identical taxonomic classification were assigned to operational taxonomic units (OTUs). To ensure equal sample sizes, all samples were subsampled to the smallest read number (41326). Prokaryotic 16s rRNA read numbers are treated as relative abundance data throughout the present study. Furthermore, OTUs representing in average less than 0.1% were summarized as “rare OTUs” and all abundance data were Box-Cox transformed before statistical analysis. Statistics and graphs were done in PAST 3 (Hammer et al. 2001) and STATISTICA 12 (StatSoft®). The analyses included non-metric multidimensional scaling using Euclidean distances as well as multivariate and univariate Permanova (9999 permutations, Euclidean distances); significances of multiple tests were corrected according to Bonferroni. All taxa of the mock community were recovered at genus level and sequencing depth was sufficient according to rarefraction. Quality filtered sequencing data has been deposited in the NCBI database as BioProject (ID: PRJNA540929).

Data analysis of physiochemical data was performed using STATISTICA 12 (StatSoft®). All experiments were deducted in three replicates. Normality of the data was tested with Shapiro-Wilk test. Significant differences were defined by one-way ANOVA, the alpha level used throughout was 0.05, and homogenous groups were calculated using Bonferroni correction.

## Results

### Addition of various SO_4_^2−^ concentrations

**Fig. 1:**
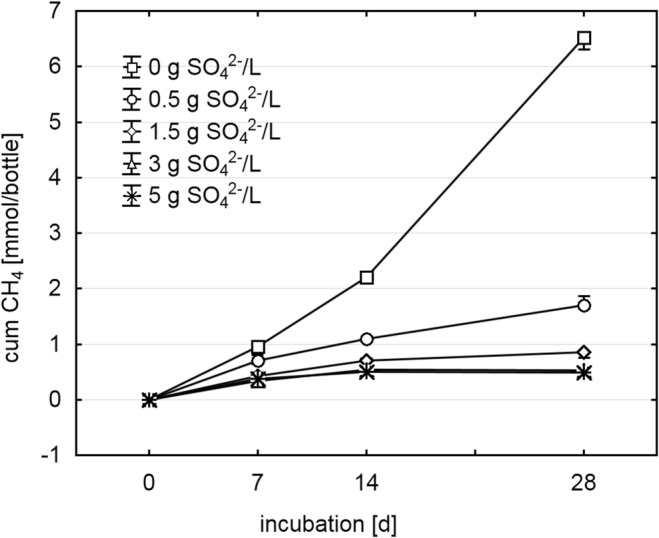
Cumulative CH_4_ production during 28 days of anaerobic digestion at various initial SO_4_^2−^ concentrations (*n* = 3, mean ± standard deviation)

**Fig. 2:**
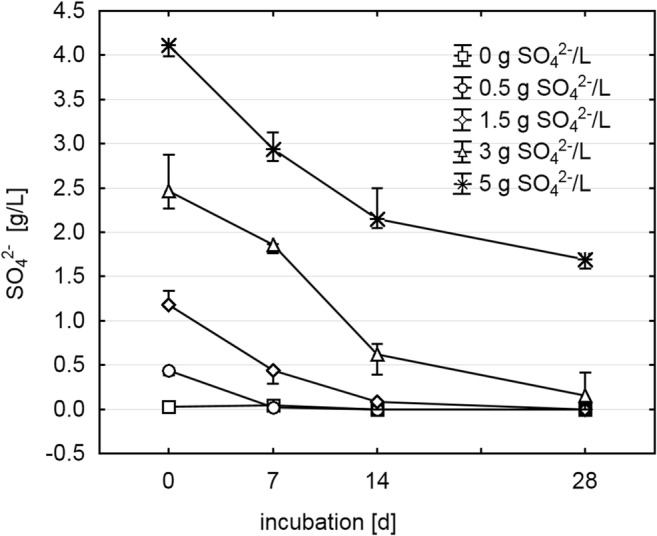
Measured SO_4_^2−^concentration during the anaerobic digestion at various initial SO_4_^2−^ concentrations (*n* = 3, mean ± standard deviation)

The COD in the inoculated medium at day 0 was 15.05 g/L, and the pH value at day 28 was 7.5 ± 0.1 over all samples. As expected, increasing initial SO_4_^2−^ concentrations of up to 3 g/L resulted in increasing inhibition of CH_4_ production, while concentrations above 3 g/L had no additional inhibitory effect during 4 weeks of incubation (Fig. 1). Cumulative CO_2_ production was slightly, but significantly, decreased in the two high-SO_4_^2−^ samples compared with the control at day 28 (0 g SO_4_^2−^/L: 6.17 ± 0.12 mmol; 3 g SO_4_^2−^/L: 4.86 ± 0.21 mmol; 5 g SO_4_^2−^/L: 4.59 ± 0.05 mmol), whereas H_2_ could not be detected at any sampling time (detection limit 0.05%). SO_4_^2−^ levels decreased throughout the incubation (Fig. 2), leading to final H_2_S concentrations in the gas phase of 0.10% ± 0.06 (0 g/L), 1.83% ± 0.17 (0.5 g/L), 3.00% ± 0.29 (1.5 g/L), 4.67% ± 0.60 (3 g/L), and 5.83% ± 0.44 (5 g/L). The viability test of methanogens and SRB, in which microorganisms from a 3 g SO_4_^2−^/L sample at day 28 were re-cultivated, was positive for both groups as 10.4% ± 0.6 CH_4_ and 4.2% ± 0.6 H_2_S could be detected after 28 days of re-incubation in fresh SO_4_^2−^-free medium and fresh SO_4_^2−^-containing medium, respectively.

### Microbial community

Illumina sequencing yielded more than 40,000 reads per sample after quality filtering and subsampling to a common read size. The microbial community of the inoculum was mainly composed by *Firmicutes* (74%), *Bacteroidetes* (8%), *Thermotogae* (6%), *Haloanaerobiaeota* (5%), and *Synergistes* (4%). At genus level, the composition of the inoculum clearly differed from all samples at the end of the experiment, with the two clostridial genera *DTU014* (20%) and *MBA03* (12%) being most abundant, followed by *Lentimicrobiaceae* (8%), *Defluviitoga* (6%), *Syntrophaceticus* (6%), *Halocella* (5%), and *Tepidimicrobium* (5%). Methanogens represented only 0.3%, while SO_4_^2−^ reducing genera were rare (< 0.1%).

**Fig. 3:**
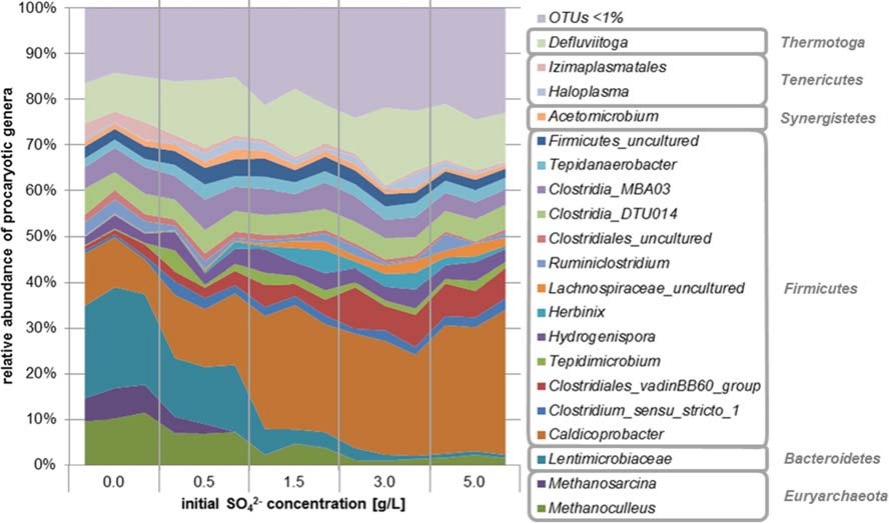
Composition of the microbial community at genus level at varying initial SO_4_^2−^ concentrations after 28 days of anaerobic incubation. Individual samples shown; OTUs comprising < 1% were summarized

After 28 days of anaerobic digestion, the microbial community was dominated by members of the phylum *Firmicutes* (45–84%), followed by *Thermotogae* (7–17%), *Bacteroidetes* (1–23%), and *Euryarchaeota* (1–18%) (Fig. 3). Less abundant phyla included *Atribacteria*, *Chloroflexi*, *Synergistes*, *Haloanaerobiaeota*, and *Tenericutes*. According to multivariate permanova analysis, the structure of the microbial consortia was significantly influenced by the initial SO_4_^2−^ concentration at all taxonomic levels. nmMDS analysis revealed that on genus level, the communities of each SO_4_^2−^ variant formed distinctive clusters mainly separated along coordinate 1, which showed a high correlation with the decrease in SO_4_^2−^ concentration in the medium during 4 weeks of anaerobic incubation (data not shown).

**Fig. 4:**
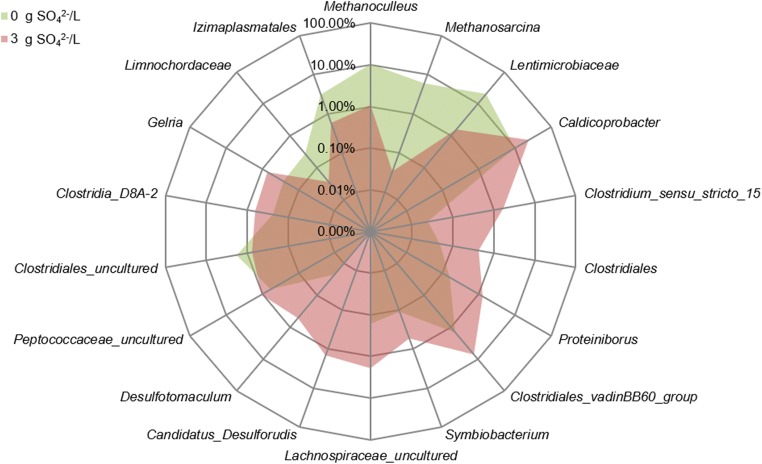
Radar chart of relative abundances [%] of genera significantly (univariate permanova, Bonferroni corrected) affected by the initial SO_4_^2−^ concentration. Means of triplicates

SO_4_^2−^ addition clearly promoted *Firmicutes* at the expense of *Bacteroidetes* and *Euryarchaeota*, whereas *Thermotogae* seemed to be unaffected by varying SO_4_^2−^ levels. At genus level, 18 of 60 OTUs with relative abundances > 0.1% showed significant differences between the SO_4_^2−^ variants (univariate Permanova, Bonferroni corrected; Fig. 4). The genera *Caldicoprobacter*, unclassified *Clostridiales_vadinBB60_group*, *Lachnospiraceae_uncultured*, *Candidatus_Desulforudis*, *Proteiniborus*, *Clostridium_sensu_stricto_15*, *Peptococcaceae_uncultured*, *Gelria*, unclassified *Clostridia_D8A-2*, *Symbiobacterium*, *Desulfotomaculum*, and unclassified *Clostridiales* (all *Firmicutes*) increased significantly with the addition of SO_4_^2−^, while unclassified *Lentimicrobiaceae* (*Bacteroidetes*), *Methanoculleus* (*Euryarchaeota*), *Methanosarcina* (*Euryarchaeota*), unclassified *Izimaplasmatales* (*Tenericutes*), unclassified *Clostridiales_uncultured* (*Firmicutes*), and unclassified *Limnochordaceae* (*Firmicutes*) were negatively influenced. The most abundant archaeal genera were *Methanoculleus* (1–11%) and *Methanosarcina* (0–7%), with the latter being completely inhibited at concentrations higher than 0.5 g SO_4_^2−^/L (Fig. 3). The most abundant bacteria were *Caldicoprobacter* (8–32%), *Defuviitoga* (7–17%), unclassified *Lentimicrobiaceae* (1–22%), unclassified *Clostridia_MBA03* (3–6%), and unclassified *Clostridia_DTU014* (4–6%), while genera known to reduce SO_4_^2−^ were found to a considerably lesser extent, comprising *Candidatus Desulforudis* (0–2%) and *Desulfotomaculum* (0–1%) (Fig. 3).

**Fig. 5:**
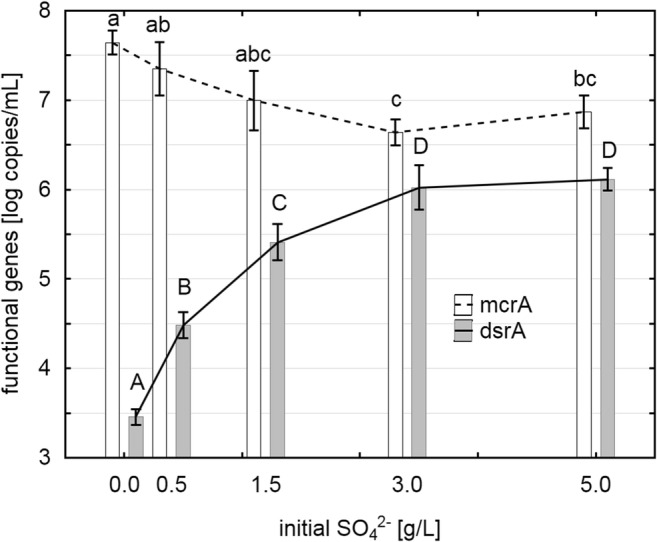
Abundance of functional genes for methanogenesis (mcrA: methyl coenzyme M reductase α-subunit) and SO_4_^2−^ reduction (dsrA: dissimilatory sulfite reductase α-subunit) at varying initial SO_4_^2−^ concentration after 28 days of incubation (mean ± standard deviation; letters show homogenous groups of ANOVA)

According to qPCR analysis, the mcrA copy number, a functional gene for methanogenesis, was up to one magnitude lower in samples containing 3 or 5 g SO_4_^2−^/L than in the controls at day 28 (Fig. 5). By contrast, the dsrA copy numbers, used to quantify SRB, increased more than 3 magnitudes with rising initial SO_4_^2−^ concentration (Fig. 5). As also seen in CH_4_ production, there was no significant difference in both functional groups between samples amended with 3 or 5 g SO_4_^2−^/L. The 1:5 diluted inoculum contained 10^6.3^ mcrA copies/mL (corresponds to 10^5.3^ copies/mL batch), while dsrA copies were below detection limit of 10^3^ copies/mL).

### Carbon flow

**Fig. 6:**
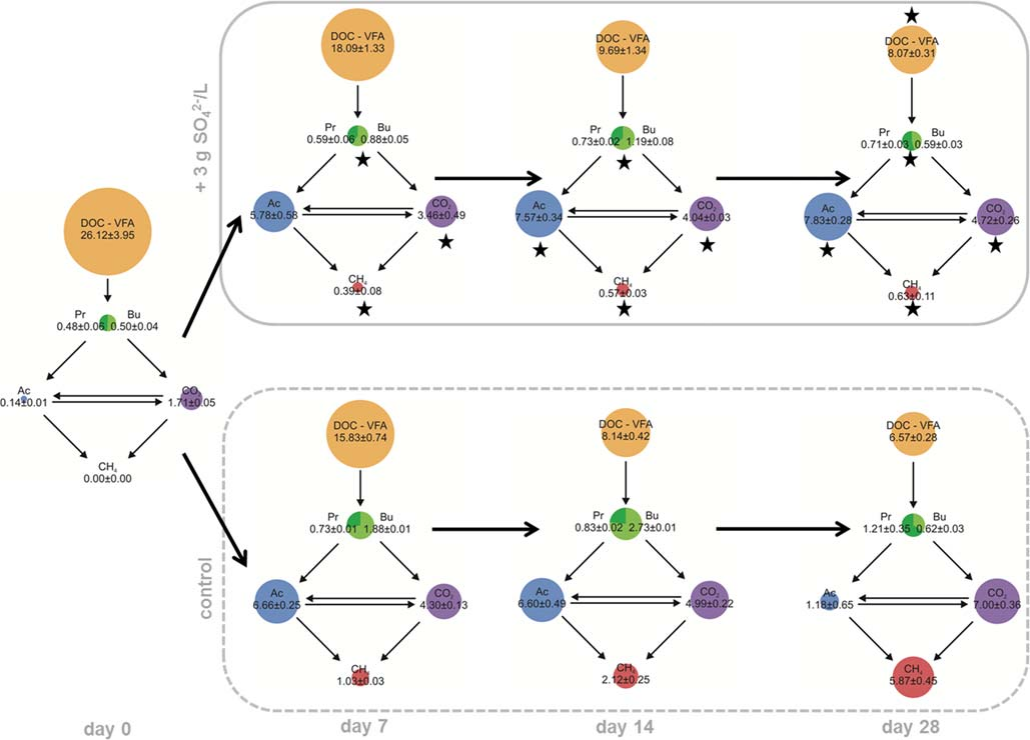
Carbon flow in samples with and without (control) 3 g SO_4_^2−^/L during 28 days of anaerobic digestion (DOC dissolved organic carbon, VFA volatile fatty acids, Pr propionate, Bu butyrate, Ac acetate). The areas of the colored circles correspond to the amount of carbon in the specific fraction. Values are given in millimoles of carbon (mean ± standard deviation). Stars depict significant differences in the specific carbon fraction between the SO_4_^2−^-samples and the controls at the same day

The carbon flow through the mixed microbial community system was calculated for controls and samples containing 3 g SO_4_^2−^/L (Fig. 6). During the first 2 weeks, concentrations of DOC decreased in both samples with and without SO_4_^2−^, leading to an accumulation of mainly acetate and CO_2_ (Fig. 6). CH_4_ production lagged behind the DOC mineralization and was significantly higher in control samples. Contrary, propionate and butyrate levels were significantly lower in SO_4_^2−^ containing samples at day 14. In the second half of the incubation, the accumulated acetate in control samples was converted to CH_4_ and CO_2_, whereas it prevailed in the in SO_4_^2−^ containing samples. At day 28, dissolved non-VFA carbon concentrations (DOC-VFA) and acetate levels were significantly higher, whereas propionate, butyrate, CH_4_, and CO_2_ concentrations were significantly lower in the SO_4_^2−^-containing samples compared with the control.

## Discussion

The addition of SO_4_^2−^ to the system affected the anaerobic digestion process on multiple levels, including gas production, VFA concentrations, the microbial community structure, and the absolute abundance of SRB and methanogens. The threshold for maximal inhibition of methanogenesis was 3 g SO_4_^2−^/L, which corresponds to a COD/SO_4_^2−^ ratio of 5. Data on inhibitory SO_4_^2−^ levels at thermophilic conditions are less available than at mesophilic conditions, but existing values are in a similar range as in our study. Siles et al. (2010) observed a total inhibition of CH_4_ production at 1.8 g SO_4_^2−^/L when fermenting glucose (6 COD g/L), McFarland and Jewell (McFarland and Jewell 1990) found that CH_4_ production decreased to 30% of the maximum at 0.8 g SO_4_^2−^/L when fermenting sucrose (10 g COD/L), and the CH_4_ content in biogas from the digestion of sugar beet molasses (12 g COD/L) was reduced by half at a SO_4_^2−^ concentration of 3 g/L (Colleran and Pender 2002).

In general, the inhibitory effect of SO_4_^2−^ addition could be caused by both toxic and competitive effects. The competition for carbon sources and electron donors between microorganisms is determined by Gibbs free energy, substrate affinities, cell numbers and growth rates, and the availability of electron acceptors (Lovley et al. 1982; Stams et al. 2005; Elferink et al. 1998; Visser et al. 1993). In the present investigation, SO_4_^2−^ was available in excess, at least in the samples containing 3 and 5 g SO_4_^2−^/L, in which it was not depleted until the end of the incubation. Initial abundances favored methanogens over SRB, as the inoculum derived from a well-performing biogas reactor with low SO_4_^2−^ levels, which was reflected by dsrA copy numbers below detection limit and a low relative abundance of SRB found during NGS analysis.

In the early phase of incubation, the main inhibitory mechanism of SO_4_^2−^ addition was probably the successful competition of SRB for H_2_, explaining the H_2_ levels below detection limit. H_2_ consumption combined with SO_4_^2−^ reduction yields more free energy than hydrogenotrophic methanogenesis, and SRB have a lower threshold for H_2_ uptake than methanogens, being 0.002 and 0.011 mbar, respectively (Lovley et al. 1982). Therefore, SRB outcompeted methanogens by decreasing the H_2_ partial pressure below the level necessary for successful methanogenesis, making thermodynamics irrelevant for the outcome of the competition as long as SO_4_^2−^ was not limited (Lovley et al. 1982). Subsequently, during SO_4_^2−^ reduction, toxic levels of H_2_S were reached (up to 6%), which enhanced the inhibition of CH_4_ production until the end of the incubation. In this context, the pH in the present study was 7.5 ± 0.1 at day 28, meaning that approximately 20% of the total dissolved sulfide should have been present as free H_2_S (Koster et al. 1986). This two-step inhibition mechanism was also suggested before (Karhadkar et al. 1987; McFarland and Jewell 1990).

The composition of the microbial community on phylum level was similar to results of other investigations of thermophilic methanogenic microbiomes. Considering all samples from day 28 regardless of the SO_4_^2−^ concentration, *Firmicutes* was the dominant prokaryotic taxon (45–84%), with comparable relative abundances also found by (De Vrieze et al. 2018; Liang et al. 2018; Lin et al. 2017; Maus et al. 2016; Tuan et al. 2014), ranging from 37 to 96%. Also, *Thermotogae*, *Bacteroidetes*, *Tenericutes*, *Synergistes*, and *Chloroflexi* were found, which are common members of a thermophilic CH_4_ producing community (De Vrieze et al. 2018; Liang et al. 2018; Lin et al. 2017; Maus et al. 2016; Tuan et al. 2014), with *Bacteroidetes* being exceptional abundant in our control samples. Generally, microorganisms can be affected by increased SO_4_^2−^ concentrations on three levels: (i) they are able to directly use SO_4_^2−^ as terminal electron acceptor, (ii) they are positively or negatively influenced by the change in abiotic conditions (including pH and substrate availability) caused by ongoing SO_4_^2−^ reduction, and (iii) they are inhibited by H_2_S or they benefit from the inhibition of competing microorganisms.

To our knowledge, no NGS data on SO_4_^2−^-influenced communities under thermophilic conditions has been available until now. Under mesophilic conditions, the dominant SRB were diverse, with *Desulfomicrobium*, *Desulfobulbus*, and *Desulfovibrio* (all *Proteobacteria*) when digesting maize silage and pig manure (Kushkevych et al. 2017); *Desulfosporosinus* (*Firmicutes*) when digesting manure (St-Pierre and Wright 2017); and *Desulfonauticus* (*Proteobacteria*) when digesting synthetic wastewater containing sucrose and ethanol under a high phenol load. In the present investigation, the candidate genus *Desulforudis* (*Firmicutes*) was most abundant, with up to 2% in samples with 3 or 5 g SO_4_^2−^/L. The description of this genus is based on the genome analysis of a single uncultivated bacterium found in a South African gold mine, as no cultivated representative is available at present (Chivian et al. 2008). Furthermore, with increasing SO_4_^2−^ concentrations, species belonging to the OTU *Desulfotomaculum* (*Firmicutes*) occurred in increasing abundances up to 1% of total prokaryotes. Until 2018, the genus *Desulfotomaculum* comprised a heterogeneous and polyphyletic group of thermophilic spore-forming SRB, which were reclassified into five genera by Watanabe et al. (2018). Since the SILVA database used in this investigation was from December 2017, the OTU “*Desulfotomaculum*” contains the present-day genera *Desulfotomaculum*, *Desulfallas*, *Desulfofundulus*, *Desulfofarcimen*, and *Desulfohalotomaculum* (Watanabe et al. 2018). Both OTUs, *Candidatus Desulforudis* and *Desulfotomaculum*, are thermophilic and are able to oxidize H_2_ among other substrates, including various sugars, VFAs, and alcohols (Muyzer and Stams 2008; Chivian et al. 2008). Besides, it should be considered that further members of the microbial community could be able to reduce SO_4_^2−^ but were not isolated or described as such this far. Especially, the family *Peptococcaceae* contains versatile physiological groups including SRB, as the two genera mentioned above (Stackebrandt 2014).

Apart from known SRB, seven bacterial OTUs were positively affected by the SO_4_^2−^ addition and showed more than 1% relative abundance at high SO_4_^2−^ levels. The most abundant of those was *Caldicoprobacter* (up to 29%), a genus, comprised of only four cultivated species, known to ferment a range of sugars including glucose and cellobiose (both intermediates of CMC mineralization) to lactate, acetate, ethanol, CO_2_, and H_2_ under thermophilic conditions (Yokoyama et al. 2010; Bouanane-Darenfed et al. 2013, 2011, 2015). Up to date, no SO_4_^2−^ reducing *Caldicoprobacter* species was found and the genus was rarely detected during NGS investigations of biogas sludge, e.g., De Vrieze et al. (2018). The OTU unclassified *Clostridiales_vadinBB60_group* was less abundant (up to 7%) and is based on a 16s rRNA gene fragment sequenced, when investigating the microbial diversity in an anaerobic digester fermenting vinasses (Godon et al. 1997). Further, positively affected OTUs include the following: the genus *Proteiniborus*, mainly known for fermenting proteins to acetate, ethanol, and H_2_/CO_2_ (Hahnke et al. 2018; Niu et al. 2008); members of the *Clostridium sensu stricto 15 group*; and uncultured members of the family *Peptococcaceae*. The latter is, as mentioned above, an ecologically and physiologically heterogeneous group of obligate anaerobes, including besides SRB also syntrophic, autotrophic as well as heterotrophic bacteria (Stackebrandt 2014).

Negatively influenced bacterial OTUs (> 1% abundance in control samples) were *Lentimicrobiaceae*, *Izimaplasmatales*, and uncultured *Clostridiales*. At the moment, the family *Lentimicrobiaceae*, representing the third abundant OTU (up to 21%) in the present study, includes only one described species, *Lentimicrobium saccharophilum*, which was isolated from a mesophilic UASB reactor, fermenting sugars to acetate, malate, propionate, formate and H_2_ (Sun et al. 2016). The order *Izimaplasmatales* belongs to the phylum *Tenericutes*, whose members are cell wall free bacteria typically found as parasites or commensals of eukaryotic hosts (Skennerton et al. 2016). The order is based on the genomes of two free-living bacteria extracted from a deep-sea CH_4_ seep sediment (Skennerton et al. 2016).

In contrast, some relatively abundant OTUs were not affected by the SO_4_^2−^ concentrations. The second most abundant OTU *Defluviitoga* comprises only one cultivated species, *Defluviitoga tunisiensis*, isolated from a mesophilic anaerobic reactor, fermenting among others cellobiose and glucose to acetate, H_2_ and CO_2_ (Hania et al. 2012). Members of the genus *Defluviitoga* have been detected in great abundances in thermophilic reactors (Liang et al. 2018; Maus et al. 2016). Further on, both OTUs *Clostridia_DTU014* and *Clostridia MBA03* showed average relative abundance of 5% and are based only on sequences isolated from thermophilic biogas reactors (Campanaro et al. 2016).

The archaeal community in the control samples consisted mainly of two methanogenic genera, *Methanoculleus* and *Methanosarcina*, comprising 10% and 6% of prokaryotic 16 s DNA, respectively. Both were inhibited by SO_4_^2−^ addition, with *Methanosarcina* being more sensitive and totally inhibited at more than 0.5 g SO_4_^2−^/L, while *Methanoculleus* preserved a relative abundance of 4% at 1.5 g SO_4_^2−^/L. The higher susceptibility towards sulfide inhibition of acetoclastic compared with hydrogenotrophic methanogens was also observed previously by Maillacheruvu and Parkin (1996).

As expected, the absolute abundance of SRB was pushed by the SO_4_^2−^ addition to 10^6^ copies/mL but surprisingly did not exceed the abundance of methanogens, even in samples with excessive SO_4_^2−^. Moestedt et al. (2013), who investigated the abundance of SRB in 25 industrial biogas fermenters with various SO_4_^2−^ loads, found 10^5^ to 10^7^ copies/mL. In our investigation, mcrA copy numbers, which were used to quantify methanogen, of more than 10^6^ could be found even in samples in which methanogenesis had been totally inhibited for at least 2 weeks, probably deriving from inactive or dead cells (Wagner et al. 2008). This result highlights the discrepancy between DNA-based abundance measurements and physiologically determined activity parameters and underlines the importance of biochemical data. To determine if the measured methanogen-specific DNA derived from free DNA, inactive cells or dead cells, a viability test was performed. It could be shown that methanogenesis restarted immediately when a small aliquot of organisms of a 3 g SO_4_^2−^/L variant was transferred into new SO_4_^2−^ free medium, meaning that inhibition by H_2_S was not lethal but reversible. The same is valid for the SRB, which also seemed inhibited by H_2_S, referring to the low CO_2_ production in week three and four of cultivation; transferred into fresh SO_4_^2−^-containing medium, SRB instantly resumed their metabolic activities.

In the present study, the addition of SO_4_^2−^ led to a decrease in propionate and butyrate concentrations and to an accumulation of acetate, although both, methanogens and SRB, should be able to utilize acetate (Muyzer and Stams 2008). This phenomenon was also observed by other authors under mesophilic, but also thermophilic conditions (O’Flaherty et al. 1998; Colleran and Pender 2002). As SRB have a lower threshold for H_2_, these organisms create a lower H_2_ partial pressure than methanogens, which improves the thermodynamics for VFA-oxidizing H_2_-producing acetogens (Stams et al. 2005). Furthermore, SRB themselves incompletely oxidize propionate and butyrate to acetate, with especially propionate oxidation being thermodynamically extremely favorable (O’Flaherty et al. 1998). Under the physiochemical conditions at day 14, the oxidation of propionate and butyrate with SO_4_^2−^ yields a ΔG of − 265 and − 94 kJ/mol, respectively. The preference of SRB for propionate over other organic acids was experimentally shown previously by Visser et al. (1993). This means, SRB directly and indirectly cause low levels of propionate and high levels of acetate. The higher the SO_4_^2−^ level, the more decisive is the direct effect. In this context, Li et al. (2015) successfully tested the application of low additions of SO_4_^2−^ (COD/SO_4_^2−^ 200 to 350) to fight propionate accumulation during a thermophilic co-digestion of coffee grounds, milk, and waste activated sludge.

The prevalence of acetate in the SO_4_^2−^ samples in the present study might be explained by the inhibition of the acetoclastic methanogenic genus *Methanosarcina* by H_2_S, as seen in the NGS results. Moreover, the SO_4_^2−^ samples were metabolically not very active in the last 2 weeks, indicating that all organisms including the SRB were at least partially inhibited by the high H_2_S concentrations. Moreover, it is possible that acetate-utilizing SRB were not present in the applied inoculum, although thermophilic acetate-oxidizing SRB have previously been isolated from methanogenic reactors (Min and Zinder 1990; Hattori et al. 2000; Balk et al. 2007). The OTU *Desulfotomaculum* comprises species able as well as species unable to oxidize acetate, while corresponding information is not available for *Candidatus Desulforudis*. These possible explanations were also mentioned by Colleran and Pender (2002), who also observed high effluent levels of acetate during thermophilic digestion of SO_4_^2−^-rich wastewater. Contrary to that, Visser et al. (1992) found that SO_4_^2−^ reducers could outcompete acetoclastic methanogens under thermophilic conditions fed on an acetate/propionate/butyrate substrate mixture.

In conclusion, our results show that SRB are able to strongly influence their abiotic environment and thus the entire microbial community even in low abundances. Finally, the altered community and chemical properties lead to a distinct shift in the carbon flow of the entire system.
